# Spatial variations in family planning demand to limit childbearing and the demand satisfied with modern methods in sub-Saharan Africa

**DOI:** 10.1186/s12978-022-01451-5

**Published:** 2022-06-22

**Authors:** Babayemi O. Olakunde, Jennifer R. Pharr, Daniel A. Adeyinka, Lung-Chang Chien, Rebecca D. Benfield, Francisco S. Sy

**Affiliations:** 1 Department of Environmental and Occupational Health, School of Public Health, University of Nevada, Las Vegas, NV USA; 2 Present Address: Department of Community Prevention and Care Services, National Agency for the Control of AIDS, 3, Ziguinchor Street, off IBB Way, Wuse Zone 4, Abuja, Nigeria; 3 Present Address: Center for Translation and Implementation Research, University of Nigeria Nsukka, Enugu, Nigeria; 4 Department of Community Health and Epidemiology, University of Saskatchewan, Saskatoon, Canada; 5 Department of Public Health, National AIDS and STI Control Programme, Federal Ministry of Health, Abuja, Nigeria; 6 Department of Epidemiology and Biostatistics, School of Public Health, University of Nevada, Las Vegas, NV USA; 7 School of Nursing, University of Nevada, Las Vegas, NV USA

**Keywords:** Demand satisfied, Fertility-limiting behavior, geographical distribution, Spatial analysis, Family planning

## Abstract

**Background:**

There is an increasing demand for family planning to limit childbearing in sub-Saharan Africa (SSA). However, limited studies have quantified the spatial variations. This study examined: (i) the spatial patterns in the demand for family planning to limit childbearing and satisfied with modern methods, and (ii) the correlates of the demand for family planning to limit childbearing satisfied with modern methods in SSA.

**Methods:**

This study analyzed secondary data on 306,080 married/in-union women obtained from Demographic Health Surveys conducted between 2010 and 2019 in 33 sub-Saharan African countries. We conducted exploratory spatial data analysis, with countries as the unit of analysis. We also performed regression analysis to determine the factors associated with demand for family planning to limit childbearing satisfied with modern methods in SSA.

**Results:**

The mean percentage of women who demanded for family planning to limit childbearing by country was 20.5% while the mean prevalence of demand for family planning to limit childbearing satisfied with modern methods by country was 46.5%. There was a significant positive global spatial autocorrelation in the demand for family planning to limit childbearing (global Moran’s I = 0.3, p = 0.001). The cluster map showed the concentration of cold spots (low–low clusters) in western and central Africa (WCA), while hot spots (high–high clusters) were concentrated in eastern and southern Africa (ESA). Also, the demand for family planning to limit childbearing satisfied with modern methods showed significant positive global spatial autocorrelation (global Moran’s I = 0.2, p = 0.004) and concentration of cold spots in WCA. In the final multivariable regression model the joint family planning decision making (β = 0.34, p < 0.001), and antenatal care (β = 13.98, p < 0.001) were the significant factors associated with the demand for family planning to limit childbearing satisfied by modern methods.

**Conclusions:**

There are significant spatial variations in the demand for family planning to limit childbearing and the demand satisfied by modern methods, with cold spots concentrated in WCA. Promoting joint decision making by partners and increasing uptake of antenatal care may improve the demand for family planning to limit childbearing satisfied with modern methods.

## Plain Language Summary

In sub-Saharan Africa (SSA), studies have shown that the proportion of married women who want to stop having children has been increasing as well as the proportion using modern contraceptive methods among them. These studies also indicated that this proportion of women are higher in certain regions of Africa than the others. To extend these previous findings, we performed geographical analysis to assess how the proportion of married/in-union women who want to stop having children and the ones using modern methods among them differ geographically. Our findings indicated that neighboring countries where the proportion of married/in-union women who want to stop having children was higher than the overall average were concentrated in eastern and southern Africa (ESA), while neighboring countries in which the proportion of married/in-union women who want to stop having children was lower than the overall average were concentrated in western and central Africa (WCA). Similarly, the results also showed that neighboring countries where the proportion of married/in-union women using modern contraceptive methods among those who want to stop having children was lower than the overall average were concentrated in WCA. Our findings suggest that increasing joint decision making on family planning and uptake of antenatal care in SSA may improve the use of modern contraceptive methods among married/in-union women who want to stop childbearing.

## Background

Contraceptive use allows individuals or couples to delay, space, or limit (stop) childbearing [1]. By preventing unintended pregnancies, contraceptive use contributes to maternal and infant survival, poverty reduction, and economic growth [2]. The use of contraceptives for family planning has been recognized as one of the 10 greatest public health achievements of the twentieth century [3], and it has continued to be featured on the global agenda for economic and social development. For example, improving access to modern contraceptive methods was one of the targets of the Millennium Development Goals (MDGs) which ended in 2015 [4]. The ongoing Sustainable Development Goals (SDGs) also specifies universal access to family planning services by 2030, with the demand for family planning satisfied with modern methods as one of the indicators for monitoring this target [5]. While there is no standardized definition for modern methods [6, 7], they have been found to be more effective than traditional contraceptive methods [8].

Clients seeking to limit childbearing are an important population that require effective contraceptive methods to prevent unintended pregnancies. In sub-Saharan Africa (SSA), where there is a rapid unsustainable population growth, prevention of unintended pregnancies among clients with the intention to limit childbearing may have an impact on fertility rates [9, 10]. Interestingly, evidence suggests increasing demand for family planning to limit childbearing in SSA [11]. For instance, in countries such as Kenya, Lesotho, Malawi, Namibia, Rwanda, and Swaziland, the demand for family planning to limit childbearing was found to exceed the demand for child spacing [12]. However, findings by Van Lith et al. indicated that a considerable proportion of limiters using contraceptives in SSA rely on traditional methods [10]; increasing their risks of having unintended pregnancies.

While evidence from descriptive studies suggests geographic variation in the demand for family planning to limit childbearing and the demand satisfied with modern methods in SSA [11, 12], to our knowledge, no prior studies have quantified these spatial relationships. Despite the growing literature on spatial dimensions of contraceptive use in SSA, available studies have focused on contraceptive prevalence of modern methods among women of reproductive age [13–21] or unmet needs [22, 23], with a majority in select countries.

Identifying spatial clusters and gaining insights into shared demographic, health systems, or economic factors by contiguous areas can inform interventions to improve uptake of family planning services among women who are seeking to limit childbearing. Accordingly, this study examined: (i) the spatial patterns in the demand for family planning to limit childbearing and the demand satisfied with modern methods and (ii) the correlates of the demand for family planning to limit childbearing satisfied with modern methods in SSA.

## Methods

### Data source and study sample

**Fig. 1:**
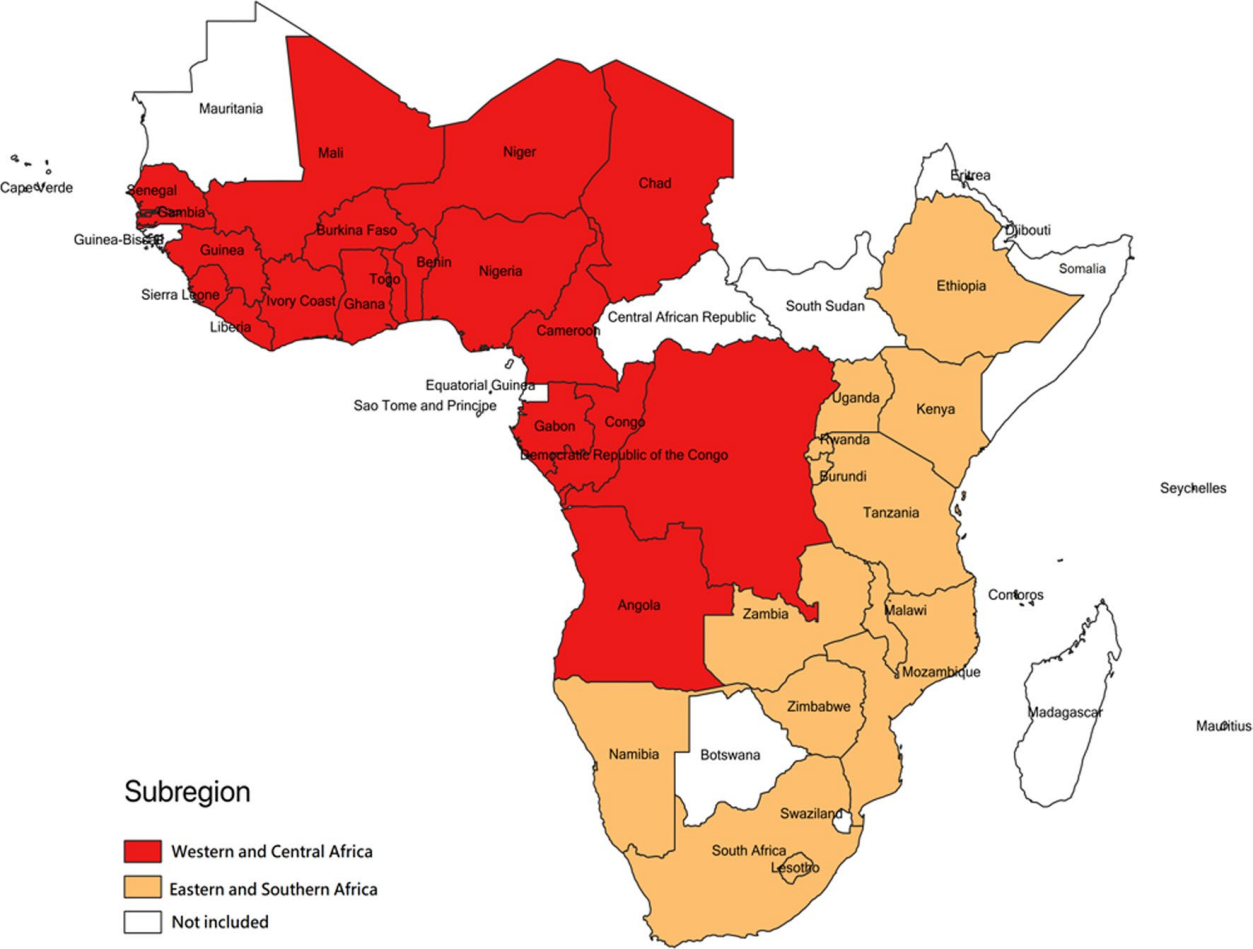
Countries included in the study by subregion

This study analyzed secondary data obtained from 33 Demographic Health Surveys [24] conducted in 33 countries and from other two data repositories (World Bank Open Data [25] and World Health Organization Global Health Observatory Data [26]). The Demographic Health Surveys (DHS) are nationally representative household surveys that gather data on several health-related topics, including family planning, in low- and middle-income countries. The methodology and procedures are standardized, making the surveys in the different countries comparable. The DHS program uses a stratified two-stage probabilistic sampling design [27]. The samples are drawn from an existing sampling frame, usually the latest census frame [27]. The sampling frame is usually stratified by geographic region and by area of residence (urban and rural) within each region [27]. The first stage involves the selection of the primary sampling units (PSU) (usually enumeration areas from population census files), with the probability of selecting a unit proportional to its size within each stratum. The second stage involves selecting a fixed number of households; about 25–30 households per PSU [27]. A detailed description of the DHS design can be found elsewhere [27]. We included 33 countries with a standard DHS conducted within the last 10 years (2010–2019) (Fig. 1). Our study sample was restricted to 306,080 married or in-union (i.e., living with a partner) women of reproductive age (15–49 years) (Table 1).

**Table 1:** Description of study sample

Subregion and country	Survey year	Number of women of reproductive age group	Number of married/in-union women of reproductive age
Western and Central Africa			
Angola	2015–16	14,379	8033
Benin	2017–18	15,928	11,170
Burkina Faso	2010	17,087	13,392
Cameroun	2011	15,426	9805
Chad	2014–15	17,719	13,439
Congo	2011–12	10,819	6750
Cote d’Ivoire	2011–12	10,060	6453
Democratic Republic of Congo	2013–14	18,827	12,448
Gabon	2012	8422	4749
Gambia	2013	10,233	6905
Ghana	2014	9396	5456
Guinea	2018	10,874	7812
Liberia	2013	9239	5875
Mali	2018	10,519	8332
Niger	2012	11,160	9509
Nigeria	2018	41,821	28,888
Senegal	2017	16,787	11,394
Sierra Leone	2013	16,658	10,754
Togo	2013–14	9480	6360
Eastern and Southern Africa			
Burundi	2016–17	17,269	9559
Comoros	2012	5329	3291
Ethiopia	2016	15,683	9824
Kenya	2014	31,079	19,036
Lesotho	2014	6621	3609
Malawi	2015–16	24,562	15,952
Mozambique	2011	13,745	8956
Namibia	2013	9176	3366
Rwanda	2014–15	13,497	6890
South Africa	2016	8514	2841
Uganda	2016	18,506	11,379
Tanzania	2015–16	13,266	8189
Zambia	2013–14	16,411	9649
Zimbabwe	2015	9955	6015
All countries	2010–18	478,447	306,080

### Measures

The DHS program collects data on the contraceptive methods currently being used by women, and report on the met and unmet needs for family planning to limit childbearing. In the survey, women are described as having: (i) met need for limiting if they are using a method of contraception and want no more children; are sterilized; or say they cannot get pregnant when asked about the desire for future children and (ii) unmet need for limiting if they are not using a method of contraception and are pregnant and did not want the current pregnancy at all; postpartum amenorrheic and did not want their last birth at all; or fecund and do not want any more children [27]. We assessed two indicators: demand for family planning to limit childbearing and the demand for family planning to limit childbearing satisfied with modern methods. We defined the demand for family planning to limit childbearing as the percentage of married/in-union women who had met or an unmet need to limit childbearing and the demand for limiting childbearing satisfied with modern methods as the percentage of married/in-union women with demand for family planning to limit childbearing using modern methods. Consistent with the DHS program, modern methods in this study included: pill, intrauterine device, injection, diaphragm, condom, male permanent contraception, female permanent contraception, implants, lactational amenorrhea, female condom, foam and jelly, emergency contraception, and standard day method [27]. For the correlates of the demand for family planning to limit childbearing satisfied with modern methods, we examined the following factors that have been found to influence the uptake of family planning methods in previous literature [28–34]: individual-level factors (educational attainment, occupation, area of residence, exposure to family planning messages on mass media, household wealth index, distance to health facility, husband/partner’s educational attainment, husband/partner’s occupation, joint family planning decision making, and antenatal care), and country-level factors (out-of-pocket expenditure, gross national income per capita, and density of nurses/midwives) (see Table 2 for the description of the explanatory variables).

**Table 2:** Description of the explanatory variables

Variable	Description	Source
Educational attainment	Percentage of married/in-union women with demand to limit childbearing with secondary or higher education	DHS
Household wealth	Percentage of married/in-union women with demand to limit childbearing from richest household^a^	DHS
Occupation	Percentage of married/in-union women with demand to limit childbearing with professional/technical/managerial job	DHS
Media exposure	Percentage of married/in-union women with demand to limit childbearing who heard about family planning in the last few months from radio, television, newspapers or magazines	DHS
Joint family planning decision making	Percentage of married/in-union women with met demand to limit childbearing who jointly made decision with their partners to use contraception	DHS
Area of residence	Percentage of married/in-union women with demand to limit childbearing who reside in urban areas	DHS
Distance to health facility	Percentage of married/in-union women with demand to limit childbearing who reported distance to health as a big problem for getting medical help	DHS
Husband/partner’s educational attainment	Percentage of husband/partner of married/in-union women with demand to limit childbearing with secondary or higher education	DHS
Husband/partner’s occupation	Percentage of husband/partner of married/in-union women with demand to limit childbearing with professional/technical/managerial job	DHS
Density of nurses/midwives	Number of nurses and midwives per 10,000 population	World Health Organization Global Health Observatory Data
Antenatal care	Percentage of women attended at least once during pregnancy by skilled health personnel for reasons related to pregnancy	World Bank Open Data
Out-of-pocket expenditure	Percentage of total current health expenditure that is out-of-pocket payment	World Health Organization Global Health Observatory Data
Gross national income per capita	The gross national income, converted to U.S. dollars using the World Bank Atlas method, divided by the midyear population	World Bank Open Data

^a^A composite measure of a household’s cumulative living standard, estimated by the survey using household’s ownership of selected assets, such as televisions and bicycles; materials used for housing construction; and types of water access and sanitation facilities. It was grouped into quintiles in DHS: Poorest, Poor, Middle, Rich, and Richest

### Statistical analysis

We conducted exploratory spatial data analysis (ESDA) with the countries as the unit of analysis in a geographic coordinate polygon shapefile of SSA [35]. The shapefile has a standard World Geodetic System 1984 (WGS84) which sets its angular units in degrees and Greenwich as the prime meridian (longitude 0 degree). We generated a spatial weights matrix using the distance band method, with the bandwidth set at an arc distance of 3000 km. The connectivity histogram indicated an even distribution of the neighbor cardinality and absence of isolates. The global Moran’s I statistic was used to assess the overall spatial autocorrelation, while the local indicator of spatial association (LISA) was used to identify the specific locations of the clusters. The LISA cluster maps showed the significant locations in four color-coded categories: low–low, high–high, low–high, and high–low. The terms low and high are defined relative to the overall mean of the indicators [36]. A low–low (or cold spot) location signified a country with a low value surrounded by countries with low values, while a high–high (or hot spot) location signified a country with a high value surrounded by countries with high values. A low–high location signified a country with a low value surrounded by countries with high values, while a high–low location signified a country with high value surrounded by countries with low values. The high–high and low–low locations (positive local spatial autocorrelation) are referred to as spatial clusters, while low–high and high–low locations (negative local spatial autocorrelation) are referred to as spatial outliers [36].

We also performed confirmatory spatial data analysis to determine the factors associated with the demand for family planning to limit childbearing satisfied with modern method. We first conducted a univariate ordinary least squares (OLS) regression analysis, and the significant variables were included in the multivariable OLS regression analysis. A backward stepwise approach was used to fit a parsimonious global multivariate model with the least number of statistically significant variables and lowest Akaike information criterion (AIC). The Lagrange Multiplier lag (LM-lag) and Lagrange Multiplier error (LM-error) tests were not significant, hence we did not proceed to conducting spatial regression [36]. In the final model, the condition number was 8.44 (less than 10), indicating the absence of multicollinearity [37]. The Jarque–Bera test for non-normality (p = 0.707) and Breusch–Pagan test for heteroskedasticity (p = 0.389) were not statistically significant. The data analysis was conducted using GeoDa v. 1.14. All analyses were considered statistically significant at p < 0.05.

## Results

### Descriptive statistics

**Fig. 2:**
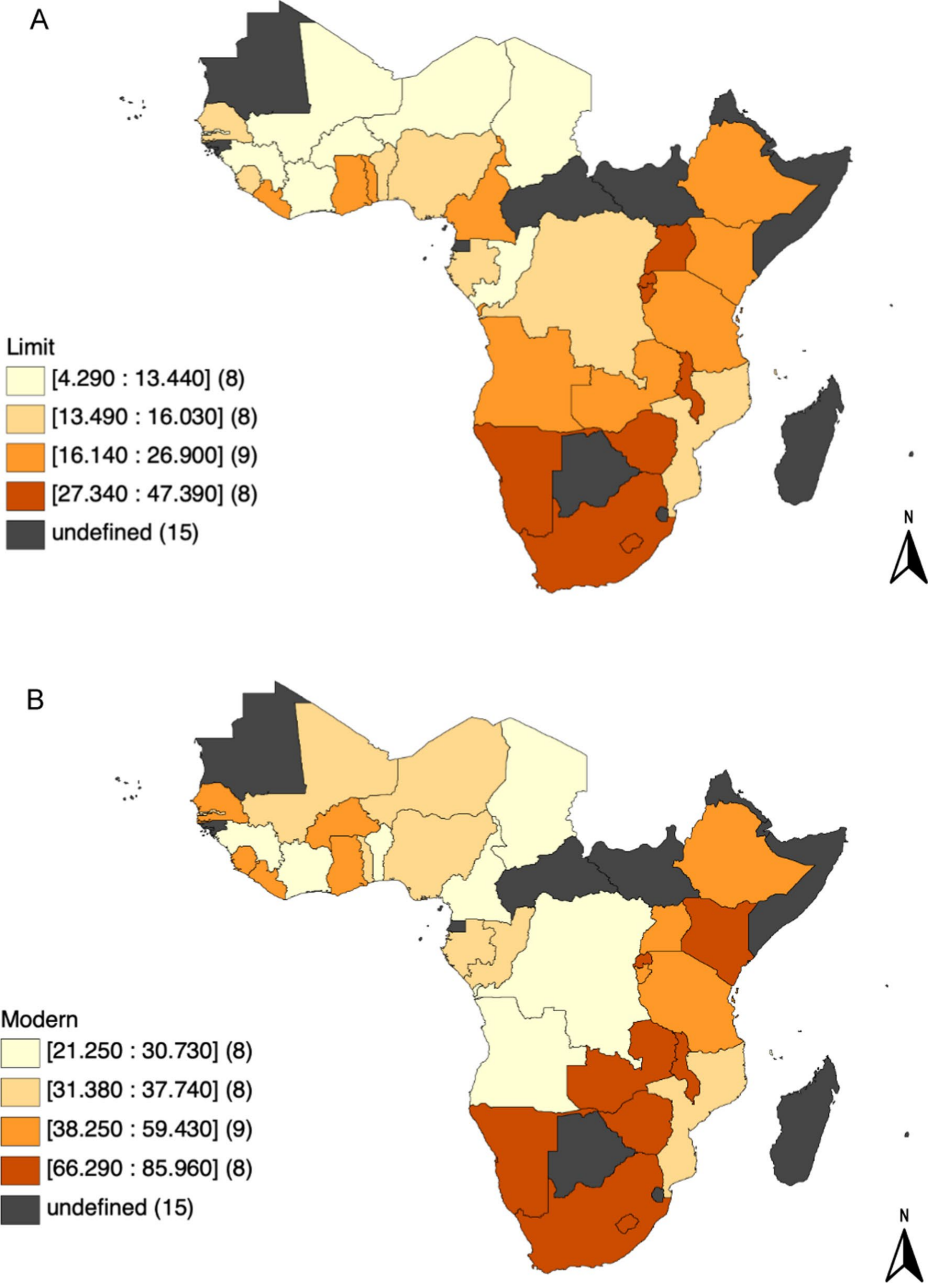
**A** Demand for family planning to limit childbearing (%). **B** Demand for family planning to limit childbearing satisfied with modern methods (%)

Table 3 shows the descriptive statistics for all the variables. The mean prevalence of demand for family planning to limit childbearing by country was 20.5%. The percentage of women who demanded for family planning to limit childbearing ranged from 4.3% in Niger to 47.4% in Lesotho (Fig. 2A). The mean prevalence of demand for family planning to limit childbearing satisfied with modern methods by country was 46.5%. The percentage of women who demanded for family planning to limit childbearing satisfied with modern methods ranged from 21.3% in Democratic Republic of Congo to 86.0% in Zimbabwe (Fig. 2B).

**Table 3:** Descriptive statistics of the outcome and explanatory variables

Variable	Mean	Standard deviation
Demand for family planning to limit childbearing (%)	20.47	11.42
Demand for family planning to limit childbearing satisfied with modern methods (%)	46.52	19.47
Educational attainment (%)	29.08	22.54
Household wealth (%)	23.73	4.25
Occupation (%)	5.49	4.01
Media exposure (%)	46.76	18.15
Joint family planning decision (%)	56.65	14.92
Area of residence (%)	41.55	17.88
Distance to health (%)	38.11	10.80
Husband/partner’s occupation (%)	11.99	5.30
Husband/partner’s educational attainment (%)	40.50	23.52
Density of nurses/midwives (per 10,000)	7.68	6.87
Antenatal care (%)	88.33	11.02
Out-of-pocket expenditure (%)	37.18	19.87
Gross national income per capita (US$)	1617.27	1919.22

### Exploratory spatial data analyses

**Fig. 3:**
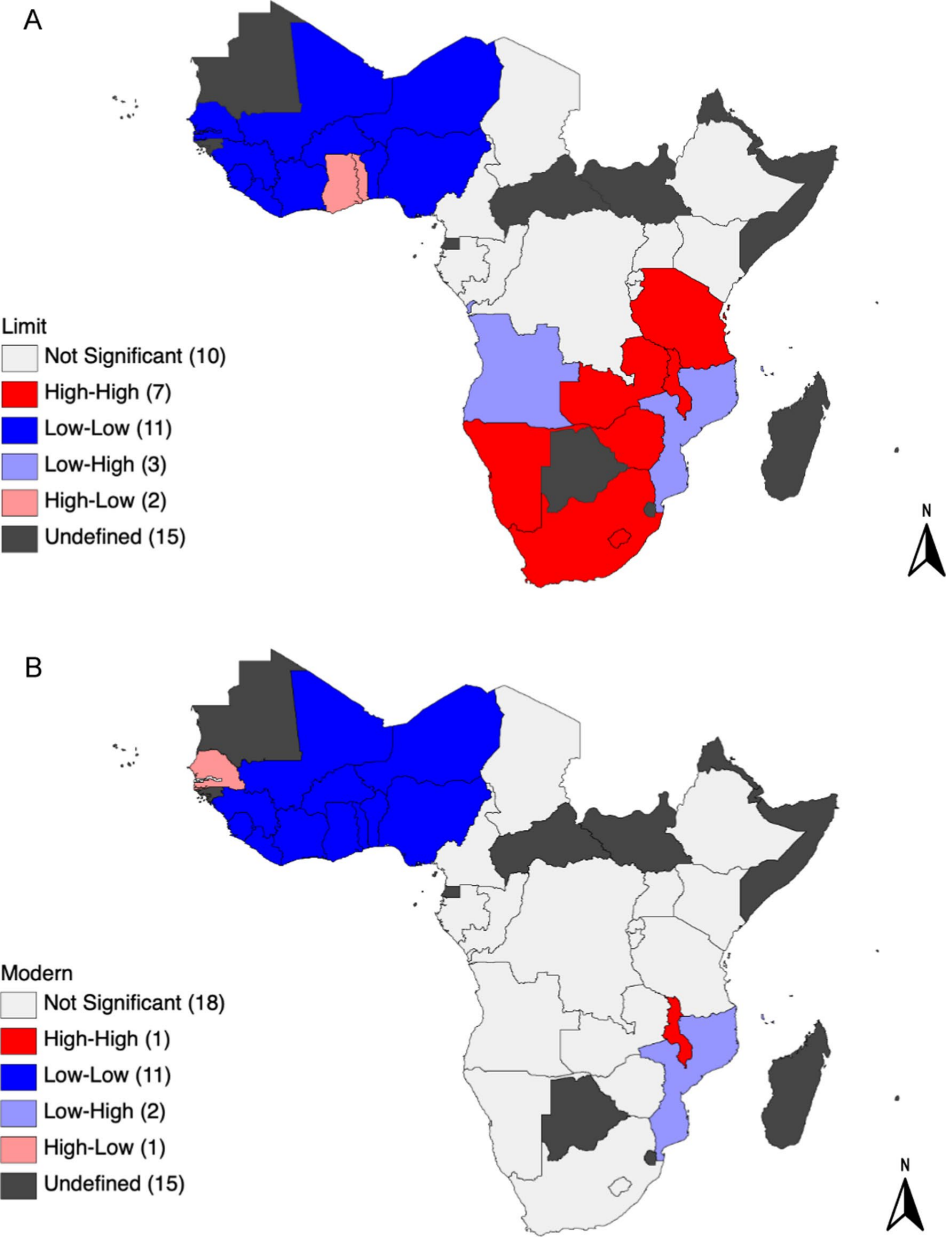
LISA cluster map. **A** Demand for family planning to limit childbearing. **B** Demand for family planning to limit childbearing satisfied with modern methods

There was a significant positive global spatial autocorrelation (global Moran’s I = 0.3, p = 0.001), indicating significant clustering of countries with similar values in the demand for family planning to limit childbearing among married/in-union women. The LISA cluster map showed that the cold spots were concentrated in WCA (Fig. 3A). These spatial clusters of demand for family planning to limit childbearing were made up of 11 neighboring countries (Benin, Burkina Faso, Cote d’Ivoire, Gambia, Guinea, Liberia, Mali, Niger, Nigeria, Senegal, and Sierra Leone) (Fig. 3A). However, there were two spatial outliers (Ghana and Togo) contiguous with the cold spots in WCA. The hot spots were found in ESA. These high–high clusters included seven neighboring countries (Lesotho, Malawi, Namibia, South Africa, Tanzania, Zambia, and Zimbabwe) with values higher than the mean (Fig. 3A). Adjacent to the hot spots were three outliers (Angola, Comoros, and Mozambique), with low demand for family planning to limit childbearing (Fig. 3A).

Also, the global spatial autocorrelation in the demand for family planning to limit childbearing satisfied with modern methods was significant and positive (global Moran’s I = 0.2, p = 0.004). The cold spots were concentrated in WCA and included 11 neighboring countries (Benin, Burkina Faso, Cote d’Ivoire, Ghana, Guinea, Liberia, Mali, Niger, Nigeria, Sierra Leone, and Togo) (Fig. 3B). However, there was one outlier of high–low (Senegal) contiguous with the cold spots (Fig. 3B). A hot spot was located in ESA (Malawi) (Fig. 3B), while there were two outliers in the sub-region (Mozambique and Comoros) with low demand for family planning to limit childbearing satisfied with modern contraceptive methods compared with their neighboring countries (Fig. 3B).

### Regression analysis

From the 13 potential independent variables, educational attainment, occupation, joint family planning decision, density of nurses/midwives, antenatal care, and out-of-pocket expenditure were significant at the univariate level (Table 4). Out of these variables, joint family planning decision making and antenatal care were selected by the backward stepwise procedure in the multivariate model (Table 5). After adjusting for antenatal care, the model showed that one unit increase in the percentage of joint family planning was associated with 0.34%-point increase in the demand for family planning to limit childbearing satisfied with modern methods (p < 0.001) (Table 5). Similarly, a unit increase in the percentage of women with antenatal care was associated 13.98%-point increase in the demand for family planning to limit childbearing satisfied with modern methods (p < 0.001) (Table 5).

**Table 4:** Univariate regression analysis of factors associated with the demand for family planning to limit childbearing satisfied with modern methods

Variable	Coefficient (β)	SE	p-value
Educational attainment	0.23	0.08	0.008
Household wealth	0.16	0.48	0.742
Occupation	1.18	0.47	0.017
Media exposure	0.10	0.11	0.391
Joint family planning decision	0.40	0.12	0.002
Area of residence	− 0.04	0.11	0.719
Distance to health	1.18	0.47	0.017
Husband/partner’s occupation	0.03	0.39	0.936
Husband/partner’s educational attainment	0.12	0.08	0.158
Density of nurses/midwives	1.00	0.24	< 0.001
Antenatal care	15.09	2.48	< 0.001
Out-of-pocket expenditure	30.76	7.03	< 0.001
Gross national income per capita	< 0.01	< 0.01	0.156

**Table 5:** Final multivariable regression analysis of factors associated with the demand for family planning to limit childbearing satisfied with modern methods

Variable	Coefficient (β)	SE	p-value
Joint family planning decision	0.34	0.07	< 0.001
Antenatal care	13.98	1.94	< 0.001
Adjusted R^2^	0.72		
AIC	215.82		
SSR	1115.11		

*AIC* Akaike information criterion, *SE *standard error, *SSR *sum of squared residual

## Discussion

The understanding of the geographic variations in the use of family planning and its determinants in SSA is important for targeted interventions to achieve the SDG target 3.7 which specifies universal access to sexual and reproductive healthcare services, including family planning by 2030. Accordingly, this study assessed the demand for and correlates of family planning to limit childbearing and the demand for family planning to limit childbearing satisfied with modern methods. The results showed significant global spatial autocorrelation, providing evidence of spatial clustering of the two indicators. On the demand for family planning to limit childbearing, the LISA map showed that cold spots were concentrated in WCA, while hot spots were concentrated in ESA. A similar pattern was observed in the demand for family planning to limit childbearing satisfied with modern methods, particularly with the concentration of cold spots in WCA. Joint family planning decision making and antenatal care were the significant factors associated with demand for family planning to limit childbearing satisfied with modern methods in SSA.

Over the years, the demand for family planning to limit childbearing has been growing in many African countries. Economic reasons, health benefits, high parity, and knowledge of family planning are some of the factors motivating or associated with the desire to limit childbearing in SSA [38–40]. However, our findings suggest that the demand for limiting varies geographically in SSA, with high–high clusters concentrated in ESA. Although there has been a long-standing debate on the relative role played by socioeconomic development and increased access to family planning on reproductive behavior in resource-limited countries [41], both factors may have accounted for the observed variation across the countries. Going by the benchmark of ≥ 75% to evaluate the demand for family planning satisfied with modern methods among those who desire to limit childbearing [42], our results suggest that several countries may be underperforming. But with strong political will and concerted efforts, immense progress can be made before 2030.

Similar to the demand for family planning to limit childbearing, the spatial pattern of the demand for family planning to limit childbearing satisfied with modern methods showed a concentration of cold spots in WCA. Prior studies have indeed demonstrated a linear relationship between the demand for family planning and demand satisfied in SSA [43, 44], suggesting that both indicators are perhaps driven by similar factors. Our results are in line with previous findings that have reported lower contraceptive use in WCA compared to ESA [45, 46], perhaps due to poorer access to family planning services. In a study that examined the reasons for contraceptive non-use among married women, the proportion of respondents who cited lack of access (including high cost, lack of source or unawareness of source to procure contraception, source too far away, and preferred method or no method available) were higher in western (9.9%) and central Africa (14.6%) than in eastern Africa (6.9%) [47]. Lower educational attainment among women and approval of family planning in western Africa have also been implicated as limiting factors in the sub-region [45].

Our results indicate that joint family planning decision has a positive effect on the demand for family planning to limit childbearing satisfied with modern methods. In many patriarchal societies in Africa, male partners play an important role regarding contraceptive use by their spouses [48–52]. Compared to limiters who made contraception decision on their own, Olakunde et al. reported that the use of female permanent contraception was higher among those who made joint decision with their partners [53]. While women’s autonomy to decision making regarding their health is important, promoting interspousal communication and male involvement may improve the coverage of modern contraception among women with demand for family planning to limit childbearing. We also found a positive relationship between antenatal care and the demand for family planning to limit childbearing satisfied with modern methods. Antenatal care presents a platform to provide family planning counselling to pregnant women [54]. However, the impact of family planning messages during antenatal care on the use of family planning has varied in literature [55–59], with evidence suggesting that frequency of antenatal care may be a moderating factor [60, 61]. The mode of counselling may also a play an important role, as uptake of family planning has been found to be higher among women who participated in group counselling during antenatal care [55]. Despite the benefits of receiving antenatal care, its uptake, particularly the recommended four or more visits remains suboptimal in SSA, especially in WCA [62, 63]. The barriers affecting antenatal care coverage in SSA are multifaceted and will require interventions at community and health system levels [64]. For women who receive antenatal care, counselling for family planning should be provided at every visit.

The study has some limitations. We included only married/in-union women, thus the findings are not generalizable to all women. The surveys we used in the study were conducted in different years, and the status of contraceptive coverage in some of the countries may have changed. Also, for some of the external variables obtained from World Bank Open Data and World Health Organization Global Health Observatory Data, the most recent available data we used did not correspond with the DHS survey year. Unavailability of information in the surveys also limited the variables considered in this study. We recommend that future spatial analysis should consider lower areal units.

## Conclusions

There are significant spatial variations in the demand for family planning to limit childbearing and the demand satisfied by modern methods in SSA, with cold spots (low–low clusters) concentrated in WCA. To improve the demand for family planning to limit childbearing satisfied by modern methods, our findings suggest the need for interventions to promote joint decision making by partners and uptake of antenatal care. As countries in SSA strive to ensure and benefit from universal access to reproductive healthcare services, it is critical that the reproductive needs of women who desire to limit childbearing are met with modern methods.

## Data Availability

Data used in study are publicly available via https://dhsprogram.com/; https://data.worldbank.org/indicator; and https://www.who.int/data/gho.

## Abbreviations

DHS: Demographic and health survey
ESA: Eastern and southern Africa
ESDA: Exploratory spatial data analysis
LIMCs: Low- and middle-income countries
LISA: Local indicator of spatial association
OLS: Ordinary least squares
SDG: Sustainable development goal
SSA: Sub-Saharan Africa
WCA: West and central Africa
